# Priorities and Understanding of Pregnancy Among Women With Congenital Heart Disease: A Mixed-Methods Study

**DOI:** 10.1016/j.jacadv.2022.100112

**Published:** 2022-10-28

**Authors:** Nicole Herrick, Tala Al-Rousan, Carla Rodriguez, Ji Hae Lee, Anne Marie Valente, Jordan Stone, Gladys Ramos, Bendelyn Asante-Boateng, Howaida El-Said, Maria Moceri-Casas, Laith Alshawabkeh

**Affiliations:** a Adult Congenital Heart Disease Program, Division of Cardiovascular Medicine, University of California-San Diego, San Diego, California, USA; b Herbert Wertheim School of Public Health and Human Longevity, University of California-San Diego, San Diego, California, USA; c Division of Cardiology, Department of Cardiology, Boston Children's Hospital, Boston, Massachusetts, USA; d Department of Medicine, Brigham and Women's Hospital, Harvard Medical School, Boston, Massachusetts, USA; e Reproductive Medicine Department, University of California-San Diego, San Diego, California, USA; f Division of Pediatric Cardiology, Rady Children's Hospital and UC San Diego School of Medicine, San Diego, California, USA

**Keywords:** adult congenital heart disease, cardio-obstetrics, mixed-methods research, pregnancy and heart disease

## Abstract

**Background:**

Many women with congenital heart disease (CHD) desire safe and successful pregnancies, but a significant proportion does not seek prepregnancy counseling.

**Objectives:**

This study aims to distinguish the personal priorities and perceptions about pregnancy in this growing population.

**Methods:**

Women aged 18 to 50 years with CHD were enrolled from 2 sites. Using a mixed-methods approach (Q-methodology), 179 participants sorted 23 statements representing a collection of views on pregnancy using priority forced ranking along a scale from “strongly agree” to “strongly disagree.”

**Results:**

Majority of women were between 25 and 29 years of age, had moderate or severely complex CHD, and were married. Five unique group identities were elucidated from patient responses. Group 1 was centered around a strong desire to start a family. Women in group 2 had significant anxiety, and their psychological wellbeing interfered with their decision to start a family. Women in group 3 were concerned about premature death; if they do have kids, they want to be alive to see them grow old. Women in group 4 had strong objections to termination. Group 5 valued health care engagement. Group identities were unrelated to CHD complexity and demographic factors such as age and marital status. Six differentiating statements were identified that help distinguish which group a woman aligns with.

**Conclusions:**

Women with CHD have diverse priorities and values relating to pregnancy and heart disease. This study used a mixed-methods approach to provide a framework identifying several domains for targeted prepregnancy counseling in women with CHD.

Women represent more than one-half of the 1.4 million adults in the United States living with congenital heart disease (CHD), many of whom hope to experience safe and successful pregnancies.^1^ Historically, many women with moderate and severe CHD were advised against pregnancy. However, with adequate surveillance and specialty care, most can safely conceive with minimal complications.^2^, ^3^, ^4^, ^5^ The increased survival and accelerated growth in the CHD population, improved quality of life, and rapid evolution in guidelines surrounding pregnancy have resulted in women receiving conflicting and often inaccurate guidance. This has resulted in a quarter of women with congenital heart disease reporting unplanned pregnancies.^6^, ^7^, ^8^ The most recent 2018 American Heart Association/American College of Cardiology guidelines for managing adults with congenital heart disease (ACHD) emphasize the role of preconception counseling to avoid unplanned pregnancy in this population.^9^

Currently, prepregnancy counseling for women with CHD often focuses on maternal peripartum cardiovascular risks; however, this counseling has significant life, career, and relational implications. Clinicians are rarely privy to these critical personal considerations because few receive specific training in pregnancy counseling.^10^^,^^11^ Understanding the personal and social values that influence a woman's decision (and invariably her health) to become pregnant can aid in providing tailored counseling to what each woman prioritizes and what is most likely to impact her long-term health, with the ultimate goal of providing individualized counseling, support, and avoidance of an unsupervised or unplanned pregnancy.

Our objective was to conduct a mixed-method research study, commonly utilized in psychology and social sciences, to assess individual “subjectivity” and to identify the spectrum of pregnancy-related values from a representative sample of women with CHD.^12^

## Methods

Women of childbearing age between 18 and 50 years, regardless of parity or gravity, with a diagnosis of CHD were prospectively enrolled using convenience sampling from May 2018 to October 2021 from multiple clinics at 2 large academic centers with comprehensive ACHD care (University of California-San Diego and Brigham and Women's/Boston Children's Hospital). After informed consent, demographic and clinical information was collected through surveys and the electronic medical record. CHD severity scores were assigned following the 2018 American Heart Association/American College of Cardiology guidelines for managing ACHD.^13^

An interview study guide was constructed after an extensive literature review. In-person unstructured interviews using this guide were conducted with key informants, and responses during these interviews served in piloting of the Q-sort and aided in refining the final draft. The Q-sort consisted of 23 statements representing a broad spectrum of views expressed around CHD and pregnancy (Table 1). Participants were required to sort all the 23 statements into a quasi-normal distribution of a 23-cell plot (Supplemental Figure 1) plotted along a numerical scale from −3 to +3, corresponding to “Strongly Disagree” through “Strongly Agree”, respectively. The Q-sort was administered with physical cards for the first 52 patients, and the remainder was completed using the Q-Sort Methodology Software during clinic visits. Unstructured feedback was elicited from participants after completing the Q-sort exercise.

**Table 1:** Q-Sort List of Statements

List of the 23 Q-sort statements	Group Ranking				
	1	2	3	4	5
My life would be incomplete without kids	+3	−1	0	1	−1
I worry about my heart condition daily	−2	+1	+1	0	−2
I would tell doctors to prioritize my own health over a fetus	0	0	0	−3	0
I would terminate an early pregnancy (<12 wks)	0	+2	+1	−3	+1
It is important I carry my own children	+2	−2	−1	+1	−1
My heart disease negatively affects my happiness	−2	+3	−2	−2	−3
I understand what has been done to my heart	+1	+3	0	+3	+3
It is important I am alive to see my kids get married	+3	+1	+3	+3	+1
I worry my doctors will tell me not to get pregnant	−1	−2	−1	−1	−1
Growing up I was told I couldn’t do many things, it turned out not to be true	−1	−1	+2	+2	+1
I frequently worry about death	−2	+2	+2	0	−3
It is important to get my doctors approval before getting pregnant	+1	+2	+3	+1	+2
I worry about my heart so much during sex I avoid it	−3	−3	−3	−2	−2
I would consider adoption	0	−1	+1	0	0
My heart disease has significant burden on my daily functioning	−3	0	−3	−2	−2
If my partner discouraged me from having kids, I would listen	−1	0	−1	−1	0
It is important to use birth control to prevent pregnancy	+2	+1	0	0	+3
I fully understand my personal risks of becoming pregnant	+1	+1	−1	+2	+2
My doctors have given me conflicting advice about pregnancy	−1	−2	+2	−1	0
My partner is fully aware of my heart condition	+1	0	0	+2	+2
I would be okay with someone else raising my child in the event of my death	0	0	−2	+1	+1
I would feel left out of my social circle if I didn’t not have kids	0	−3	−2	−1	−1
It is important that my partner wants kids	+2	−1	+1	0	0

The Q-methodology uses priority forced ranking to identify participants who ranked statements similarly and calculates a factor loading score.^12^^,^^14^ The higher the factor loading score is, the more a participant identifies with a particular group (or *factor group*). The analysis intentionally groups participants based on responses to the Q-sort and not by demographic data.

Statistical analysis was performed using the Q-method software, validated in other studies.^15^ Per previously published literature, 5 centroid factors were extracted, and 5 factors (referred to as groups from here on) were selected for varimax algorithm rotation.^14^^,^^15^ After rotational analysis, an individual participant's factor loading was considered significant at *P* < 0.05. A manual review followed this; participants loaded significantly onto more than 1 factor or did not load significantly on any factor were excluded. Weighted Z-scores were calculated to identify the distinguishing statements for each group. Categorical variables were summarized with frequencies and percentages, and continuous variables with median and standard deviation unless otherwise indicated. Summary statistics were calculated in SPSS (version 26, IBM).

The study was approved by the Institutional Review Board of the University of California San Diego, Boston Children’s Hospital, and Brigham and Women’s Hospital. The study procedures complied with Health Insurance Portability and Accountability Act regulations and obeyed the tenets put forth by the Declaration of Helsinki.

## Results

A total of 179 women aged 18 to 50 years with CHD were enrolled. All participants who gave consent completed the Q-sort. More than three-quarters of participants were younger than 40 years, and the vast majority of participants had moderate or severely complex CHD and a history of cardiac surgery (Table 2). Two-thirds were married or in a serious relationship. Forty-two percent of women had been pregnant at least once, and 35% had given birth. Approximately one-third of women who gave birth had a cesarean delivery, most of whom reported that their provider believed operative delivery was safer given their underlying heart disease (Table 3). Participant responses generated 5 unique group identities, which explained 56% of the cumulative variance.

**Table 2:** Demographic Characteristics

		Group						
		1	2	3	4	5		
		Strong Desire to Start a Family (n = 55)	Psychological Distress (n = 20)	Maximizing Longevity (n = 12)	Strongly Against Termination (n = 20)	High Health Care Engagement (n = 50)	No Single Group Identity (n = 22)	All (N = 179)
Age	*P* = 0.735							
18-24		11 (20)	3 (15)	0 (0)	3 (15)	9 (18)	2 (9.1)	27 (15.2)
25-29		17 (30.9)	5 (25)	3 (25)	3 (15)	9 (18)	8 (36.4)	45 (25.3)
30-34		8 (14.5)	6 (30)	4 (33.3)	5 (25)	14 (28)	4 (18.1)	41 (23)
35-39		10 (18.2)	3 (15)	2 (16.7)	2 (10)	9 (18)	4 (18.1)	30 (16.9)
40-50		9 (16.4)	3 (15)	3 (25)	7 (35)	9 (18)	4 (18.1)	35 (19.7)
Marital status	*P* = 0.100							
Single		15 (27.3)	5 (25)	1 (8.3)	4 (20)	19 (38)	11 (50)	55 (30.7)
Relationship		12 (21.8)	3 (15)	5 (41.7)	2 (10)	7 (14)	1 (4.5)	30 (16.8)
Married		25 (45.4)	11 (55)	5 (50)	14 (70)	24 (48)	10 (45.5)	90 (50.3)
Divorced		3 (5.5)	1 (5)	0 (0)	0 (0)	0 (0)	0 (0)	4 (2.2)
Race/Ethnicity	*P* = 0.05							
African American		4 (7.3)	1 (5)	0 (0)	1 (5)	0 (0)	2 (9.1)	8 (4.5)
Asian		6 (10.9)	0 (0)	0 (0)	1 (5)	7 (14)	1 (4.5)	15 (8.4)
Hispanic		10 (18.2)	2 (10)	3 (25)	1 (5)	4 (8)	5 (22.7)	25 (14)
White		35 (63.6)	16 (80)	9 (75)	13 (65)	38 (76)	13 (59.1)	124 (69.3)
Other		0 (0)	1 (5)	0 (0)	4 (20)	1 (2)	1 (4.5)	7 (3.9)
Mean number of pregnancies	*P* = 0.002							
		1.3 ± 1.6	0.7 ± 0.8	0.7 ± 0.8	2.1 ± 1.6	0.6 ± 1.3	0.8 ± 1.5	1.0 ± 1.5
Mean number of births	*P* = 0.007							
		0.8 ± 1.1	0.6 ± 1.1	0.3 ± 0.7	1.5 ± 1.2	0.5 ± 0.9	0.5 ± 1.1	0.7 ± 1.1
CHD complexity	*P* = 0.091							
Simple		13 (23.6)	2 (10)	0 (0)	0 (0)	4 (8)	3 (13.6)	22 (12.3)
Moderate		28 (50.9)	10 (50)	5 (41.7)	10 (50)	23 (46)	12 (54.5)	88 (49.2)
Great complexity		14 (25.5)	8 (40)	7 (58.3)	10 (50)	23 (46)	7 (31.8)	69 (38.5)
Number of sternotomies	*P* = 0.578							
0		15 (27.3)	5 (25)	3 (25)	4 (20)	8 (16)	5 (22.7)	40 (22.3)
1		21 (38.2)	8 (40)	2 (16.7)	6 (30)	13 (26)	5 (22.7)	55 (30.7)
2		9 (16.4)	1 (5)	3 (25)	3 (15)	9 (18)	6 (27.3)	31 (17.2)
3+		10 (18.1)	6 (30)	4 (33.3)	7 (35)	20 (40)	6 (27.3)	53 (29.1)

Values are n (%) or mean ± SD.

CHD = congenital heart disease; SD = standard deviation.

**Table 3:** Details of Gravity, Parity, Preconception Counseling, and Delivery Recommendations by Group Identification

		Group						
		1	2	3	4	5		
		Strong Desire to Start a Family (n = 55)	Psychological Distress (n = 20)	Maximizing Longevity (n = 12)	Strongly Against Termination (n = 20)	High Health Care Engagement (n = 50)	No Single Group Identity (n = 22)	All (N = 179)
Received advice not to become pregnant	*P* = 0.003	9 (16.4)	9 (45)	7 (58.3)	10 (50)	23 (47.9)	10 (50)	68 (38.9)
Pregnancies	*P* = 0.029							
Nulligravida (G0)		27 (49.1)	15 (75)	6 (50)	4 (20)	35 (71.4)	15 (71.4)	102 (57.6)
Primigravida (G1)		9 (16.4)	0 (0)	4 (33.3)	3 (15)	5 (10.2)	1 (4.8)	22 (12.4)
Multigravida (G2+)		19 (34.5)	5 (25)	2 (16.7)	13 (65)	9 (18.3)	5 (23.9)	53 (30)
Births	*P* = 0.028							
Nulliparous (P0)		30 (54.5)	15 (75)	9 (75)	7 (35)	37 (74)	17 (77.3)	115 (64.2)
Primiparous (P1)		10 (18.2)	1 (5)	2 (16.7)	2 (10)	5 (10)	1 (4.5)	21 (11.7)
Multiparous (P2+)		15 (27.2)	4 (20)	1 (8.3)	11 (55)	8 (16)	4 (18.1)	43 (24)
Planned pregnancy	*P* = 0.115							
No		9 (32.1)	1 (20)	4 (66.7)	7 (43.8)	2 (15.4)	1 (16.7)	24 (32.4)
Yes, met w MD		9 (32.1)	0 (0)	1 (16.7)	6 (37.5)	7 (53.8)	4 (66.7)	27 (36.5)
Yes, did not meet w MD beforehand		10 (35.7)	4 (80)	1 (16.7)	3 (18.8)	4 (30.8)	1 (16.7)	23 (31.1)
Delivery method (for first pregnancy)	*P* = 0.094							
Vaginal delivery		19 (76)	5 (100)	1 (33.3)	8 (61.5)	5 (41.7)	2 (40)	40 (63.5)
Cesarean delivery		6 (24)	0 (0)	2 (66.7)	5 (38.5)	7 (58.3)	3 (60)	23 (36.5)
My doctor said cesarean was safer for my heart		3 (50)	NA	1 (50)	4 (80)	2 (28.6)	3 (100)	13 (56.5)

Values are n (%).

### Group 1: a strong desire to start a family despite CHD

Women who identified in this group felt strongly about carrying a child themselves (less likely to consider adoption), finding a partner who also wants children, and being alive to see their children get married. The defining statement for this group is, “My life would be incomplete without children.” They feel neutral about physician approval before conception. They exhibited no significant anxiety related to their CHD, nor was it a burden on daily life. Approximately one-half of the participants have been previously pregnant and given birth. Interestingly, this group was the most likely to have received advice against pregnancy. One woman who identified with this group shared, “I consider myself fortunate and look forward to sharing information with my adult daughter who also has a congenital heart condition so she can make informed decisions.”

### Group 2: psychological wellbeing is negatively impacted by CHD

Women who identify with this group have a good understanding of their CHD and previous interventions but overwhelmingly feel that CHD is a significant burden on their daily life and negatively affects their feelings of happiness to the point where they frequently worry about death. They do not feel strongly about having or carrying their own children. They feel strongly that physician approval is essential before attempting pregnancy, and if personal health is at risk, they would consider termination. The majority of women (75%) had never been pregnant or given birth. One woman in this group shared, “I really haven’t heard much about it being dangerous for me to get pregnant, and I am questioning the seriousness of it and the state of my condition now that it has been brought to my attention.”

### Group 3: emphasize longevity but have inadequate knowledge about pregnancy risk

Women who identify with this group worry about premature death. They expressed that they have received conflicting advice from doctors about pregnancy in the setting of their CHD, and as a result, they feel that they do not have a good understanding of the risks of becoming pregnant. They feel neutral about having kids and would consider early termination if their health is at risk. However, they feel very strongly that if they have kids, they want to be alive long enough to see them grow up and get married. In contrast to group 2, they do not feel that CHD is a burden on their daily functioning, nor does it negatively affect their psychological wellbeing or happiness. One-half of the women had been pregnant, and a quarter had given birth previously. One woman in this group shared, “I feel like women that have CHDs don’t get the same answers from doctors when it comes to pregnancy.”

### Group 4: strong objection to termination of pregnancy

Women who identify with this group would not consider termination regardless of gestational age, even if their health was at risk. They feel neutral to slightly positive about having and carrying their children and endorse having a good understanding of the cardiovascular risks of pregnancy. Previous pregnancy and birth strongly distinguish this group from others. However, only one-third who identified with this group and had given birth received preconception counseling. A woman in this group shared, “This is a great study that I hope provides comfort for women to make a better plan for their future whether they decide to have children or not. I would press that the overall health is taken into account.”

### Group 5: need for empowerment and continuous health care engagement

Women who identify with this group endorse an in-depth understanding of their CHD. Through continuous health care engagement, they know what procedures have been done to their heart and the health risks associated with pregnancy. They accentuate the importance of physician approval before attempting pregnancy and emphasize the role of birth control when trying to prevent pregnancy. Therefore, CHD is not a burden on their daily life, nor does it negatively affect their happiness. The majority had never been pregnant or given birth. A woman in group 5 shared, “I personally will not be having children. The risks involved sealed the deal when going over the current studies out there and the higher risk factors that would involve me being pregnant.”

### Group and site comparisons

Age, marital status, race/ethnicity, and CHD complexity were not significantly associated with any group. There were significantly more women who had a pregnancy and delivery in groups 1 and 4 than in the other groups. There were no differences between the groups regarding planned vs unplanned pregnancy (*P* = 0.12). In total, 38% of women reported they had been told at some point in their lives that they should not become pregnant, but none had a diagnosis with an absolute contraindication to pregnancy.

Of the 179 enrolled, 157 women loaded significantly with one of the 5 groups; the remaining 22 did not significantly load in any of the 5 groups (n = 16) or loaded significantly into more than one group (n = 6). These results were validated using the Q-method software and a separate manual, iterative review. There were no significant sociodemographic differences between women who did and did not fit into a specific group identity.

There were no significant differences between the 2 sites regarding age, CHD complexity, or group identification (Supplemental Table 1). However, site A had significantly more non-Caucasian patients, and site B had more patients who were married or in a committed relationship. Site-specific analysis yielded similar results to the combined analysis, except that site B analysis yielded no group identity driven by anxiety.

Six differentiating statements that quickly helped determine the group identity were identified (Table 4, Central Illustration).

**Table 4:** Distinguishing Statements for Each of the Factor Groups and Sample Survey to Help With Rapid Identification of Group Type

Distinguishing Statement	Group 1	Group 2	Group 3	Group 4	Group 5
My life would feel incomplete without having children	z-score +1.7 [+3]	z-score −0.72 [−1]	z-score 0.01 [0]	z-score +0.59 [1]	z-score −0.91 [−2]
My heart condition negative affects my happiness	z-score −1.2 [−2]	z-score +1.2 [+3]	z-score −0.61 [−1]	z-score −0.89 [−2]	z-score −1.47 [−3]
I fully understand the risks of becoming pregnant with my heart condition	z-score 0.39 [+1]	z-score +0.87 [+1]	z-score −0.9 [−2]	z-score 1.23 [+2]	z-score 1.01 [+2]
I would terminate a pregnancy if my personal health was at risk	z-score 0.02 [0]	z-score 0.88 [+2]	z-score 0.67 [+1]	z-score −2.07 [−3]	z-score 0.73 [+1]
It is important that I get approval from my doctors before getting pregnant	z-score 0.81 [+1]	z-score 1.15 [+2]	z-score −1.51 [+2]	z-score 0.69 [+1]	z-score 1.3 [+2]
When trying to avoid pregnancy it is important to use birth control	z-score 1.01 [+2]	z-score 0.73 [+1]	z-score 0.2 [0]	z-score 0.0 [0]	z-score 1.5 [+3]
On at least one occasion I have received conflicting advice about becoming pregnant	z-score −0.61 [−1]	z-score −1.57 [−2]	z-score 1.33 [+2]	z-score −0.38 [−1]	z-score −0.11 [0]

**Central Illustration:**
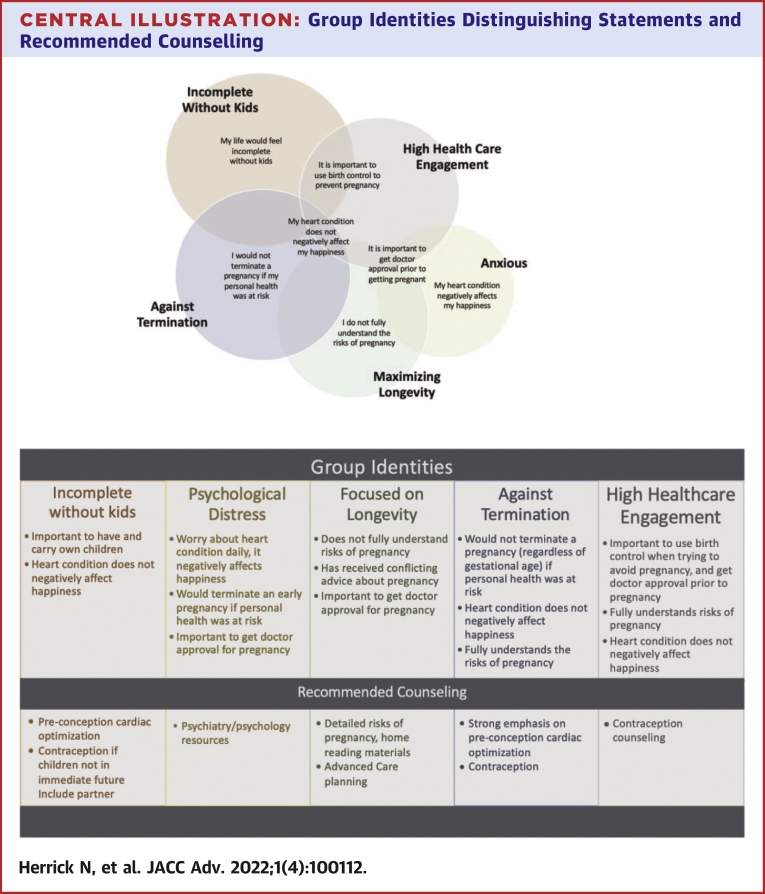
Group Identities Distinguishing Statements and Recommended Counselling

## Discussion

With the growing population of women with CHD comes a growing need for specialized combined cardiovascular and obstetric care. This study is among the first to assess perspectives and experiences of women with CHD relating to pregnancy and childbearing, intending to streamline the process of providing individualized counseling on topics most relevant to each woman. Here we provide a categorization system within the heterogeneous patient population of women with CHD.

We identified 5 distinct groups and provided tools that providers can use to identify individual priorities to facilitate the planning and preconception discussions. Lessons from this study emphasize that the depth and breadth of priorities and understanding of pregnancy in women with CHD are far more complex than can be explained with basic demographic factors like age and marital status. The first group identity is centered around a strong desire to have and carry their own children, the second is burdened by their CHD and battling with anxieties related to it, the third group is focused on longevity and wanting to grow old with their children, the fourth is firmly against termination even if personal health is at risk, and the fifth values being engaged in care and stresses the importance of preconception physician approval.

Six statements were identified as distinguishing, the answers to which facilitate identification of which group identity a woman's priorities most closely align with, free of demographic bias. For example, for a woman who identifies with group 1 and has a strong desire for a family and carrying a child herself, limited clinic time would be well spent discussing the importance of cardiac optimization before conception and reinforcing the importance of avoiding an unplanned pregnancy. It would also allow for referrals for preconception counseling by a maternal-fetal medicine physician. Notably, 20% of women within this group were between 18 and 24 years of age, and 27% were single, further highlighting the importance of not using basic criteria such as age and marital status to determine the appropriate time to discuss pregnancy and contraception. Women in this group prioritize a partner who also wants children, so it is essential to include the partner (if they have one) in counseling.

In comparison, women who identify with group 2 are driven by anxiety; thus, it would be important to spend more time ensuring they have appropriate mental health support rather than solely reviewing the risks of pregnancy. The third group is focused on optimizing longevity; however, they do not feel like they have a good understanding of the risks of pregnancy and have received conflicting advice in the past. Thus, counseling for group 3 must include a thorough explanation of risks and advanced care planning.^16^ This group felt neutral on having children, so they may benefit from a more-frank risk-benefit discussion on pregnancy to allow the individual to decide to maximize their overall health and longevity.

Groups 4 and 5 both endorsed a high-level understanding of their CHD and partner involvement; however, they diverged on the distinguishing statements. Group 4 was distinguished by a strong objection to termination, and group 5 focused on high health care engagement. While there are similarities between the groups, viewed from a clinical perspective, focusing on the differentiating factors may be useful when devising a counselling strategy. For example, if a woman who identified with group 4 desires pregnancy, it would be critical to provide in-depth preconception counselling (including clear guidelines on timing and specifics of cardiac optimization) as well as inquiry about the rationale behind the objection to termination (cultural, faith-based, misconception, personal, etc). Thus, emphasizing the distinguishing statements is more likely to yield more effective and open-ended inquiry and adequate counseling.

Many women indicated they had either an unplanned pregnancy or no preconception counseling before a planned pregnancy (33.8% and 32.4%, respectively). Interestingly, there were no between-group differences (*P* = 0.115) in preconception counseling, highlighting the need to make pregnancy planning a routine part of ACHD care for all patients, regardless of their group identity. Women currently in their 30s and 40s have seen a dynamic shift in ACHD and pregnancy guidelines throughout their lifetime. In fact, 38% of women reported they had received advice not to become pregnant although, by current guidelines, none had a diagnosis consistent with an absolute contraindication to pregnancy. Receiving conflicting advice makes it difficult to decide which to follow and may lead to avoidance of care, as seen by the relatively high number of women who reported having a planned pregnancy but did not meet with a physician prior. Additionally, one-third of women had unplanned pregnancies, which speaks to the importance of contraception counseling as part of routine ACHD care. Despite the advice to avoid pregnancy, 27 of the 68 women became pregnant.

We observed between-site differences in anxiety and the burden of CHD on daily life. Most patients enrolled from site B were in the pediatric setting, which may have impacted the patient’s understanding of CHD, self-care, and subsequent anxiety and CHD as a burden on daily life. The subtle differences in group identities between the 2 sites further highlight the potential benefits of using the distinguishing statements as a tool to enhance patient-centered care.

The findings are equally relevant to those working in pediatric and adult cardiology. Many women begin to develop opinions and priorities relating to having children at a very young age, which invariably impacts other critical life decisions, including selecting a life partner and career choices. For this reason, this conversation must begin before the transition to adult care, which often occurs between the ages of 18 and 21 years. We hope that utilization of the distinguishing statements can facilitate what may otherwise be an uncomfortable discussion regarding pregnancy in the pediatric setting. Indeed, 15% of women whose group identity was driven by objection to termination were between the ages of 18 and 24 years; therefore, it would be necessary to discuss at a young age the importance of birth control as a means to prevent unintended pregnancy.

Supplemental Table 2 is an excerpt of comments elicited after participation, highlighting the fact that the topic of pregnancy and childbearing may be very emotionally charged. Many women shared that they were happy this topic was being explored and were eager to hear the study results. This highlights the opportunity to improve patient expectations and, eventually, clinical outcomes.

### Study Limitations

Despite being one of a handful of studies examining the experiences and perspectives of women with CHD, our study has limitations. Q-methodological studies are designed to have a relatively small sample size,^17^ with the goal being to provide an in-depth understanding of the specific population in question rather than a purely quantitative assessment. However, it is possible that the relatively large sample size used in this study increased the likelihood of type-II error. Age may have been a factor in our results, as all women were older than 18 years, so our study cannot be generalizable to younger women of the childbearing age. Education level may also have contributed to a woman’s decision regarding childbearing and pregnancy, but was not assessed as a part of the current study. The 23 statements that comprised the Q-sort were created after in-person interviews with women with CHD and experts in the field to represent a full concourse of opinions on the topic of pregnancy and ACHD. However, it is possible that these 23 statements did not encompass the full spectrum of opinions, potentially excluding some from a factor group or being placed into an incorrect group. When selecting our sample utilizing convenience sampling from 2 sites, selection and channeling biases may have occurred.^18^ A woman's responses may change over time, so future research is needed to replicate study findings in other settings and evaluate temporal changes in the same group.

## Conclusions

In this cohort of 179 women of childbearing age with CHD, we found 5 unique group identities with diverging opinions and priorities relating to pregnancy and childbearing, which can help inform individual clinicians as well as public health interventions to improve the experiences of women with CHD that consider becoming pregnant. Traditional demographic factors, including age and marital status, were not predictive of opinions relating to pregnancy. Six of the 23 statements were identified as distinguishing; the answers can help identify which group identity a woman aligns most closely with, free of demographic bias, and provide a framework for clinicians to identify efficiently, in an emotionally sensitive manner, what an individual woman prioritizes to determine appropriate timing for referral to maternal-fetal medicine and improve patient-centered care.

PERSPECTIVES **COMPETENCY IN PATIENT CARE AND PROCEDURAL SKILLS:** Q-methodology was used to identify 5 unique group identities among women with CHD, each with diverging opinions and priorities relating to pregnancy (strong desire for children, CHD negatively impacting psychological well-being, concern about longevity, objection to termination, and high health care engagement). Traditional demographic factors including age and marital status were not predictive of group identity. Six statements were identified as distinguishing, the answers to which can provide a framework for clinicians to provide targeted prepregnancy counseling.

**TRANSLATIONAL OUTLOOK:** Future steps include development of a tool kit that can be widely used to streamline the process of group identification.

## Funding support and author disclosures

Dr Alshawabkeh is supported by the 10.13039/100000968American Heart Association Career Development Award grant. Dr Al-Rousan is supported through a grant from the 10.13039/100000050National Heart, Lung and Blood Institute (#K23HL148530). Ms Lee, Dr Rodriguez, and Dr Valente are supported by the Sarah Marie Liamos Fund for Adult Congenital Heart Disease Research. All other authors have reported that they have no relationships relevant to the contents of this paper to disclose.

## Footnotes

Appendix For a supplemental figure and tables, please see the online version of this paper.
