# Supplementary material for: Priorities and Understanding of Pregnancy Among Women With Congenital Heart Disease: A Mixed-Methods Study
Source: JACC Adv. 2022 Oct 28;1(4):100112. doi: 10.1016/j.jacadv.2022.100112 (PMC11198376; doi:10.1016/j.jacadv.2022.100112)
Supplement: Supplemental Figure 1 and Tables 1, 2 [file mmc1.docx]

**Supplemental Figure 1.** Sample Q-sort distribution. Each of the 23 statements are placed into one of the boxes with forced ranking.

**Supplemental Table 1.** Comparison between site A and B.

|  |  |  |  |
| --- | --- | --- | --- |
|  |  | **Site A** | **Site B** |
|  |  | n=129 | n=50 |
| Age | p=0.122 |  |  |
| 18 – 24 |  | 25 (19.4) | 3 (6) |
| 25 – 29 |  | 32 (24.8) | 13 (26) |
| 30 – 34 |  | 31 (24) | 10 (20) |
| 35 – 39 |  | 18 (14) | 12 (24) |
| 40 – 50 |  | 23 (17.8) | 12 (24) |
| Marital Status | p=0.005 |  |  |
| Single |  | 48 (37.2) | 7 (14) |
| Relationship |  | 21 (16.3) | 9 (18) |
| Married |  | 59 (45.7) | 31 (62) |
| Divorced |  | 1 (0.8) | 3 (6) |
| Race/Ethnicity | p=0.003 |  |  |
| African American |  | 7 (5.4) | 1 (2) |
| Asian |  | 13 (10.1) | 2 (4) |
| Hispanic |  | 25 (19.4) | 0 (0) |
| White |  | 78 (60.5) | 46 (92) |
| Other |  | 6 (4.7) | 1 (2) |
| CHD Complexity | p=0.842 |  |  |
| Simple |  | 17 (13.2) | 5 (10) |
| Moderate |  | 63 (48.8) | 25 (50) |
| Great Complexity |  | 49 (38) | 20 (40) |
| Sternotomies | p=0.878 |  |  |
| 0 |  | 26 (20.2) | 14 (28) |
| 1 |  | 41 (31.8) | 14 (28) |
| 2 |  | 22 (17.1) | 9 (18) |
| 3+ |  | 40 (31) | 13 (26) |
| Group Identity | p=0.536 |  |  |
| 1 |  | 35 (31.5) | 20 (43.5) |
| 2 |  | 14 (12.6) | 6 (13) |
| 3 |  | 10 (9) | 2 (4.3) |
| 4 |  | 16 (14.4) | 4 (8.7) |
| 5 |  | 36 (32.4) | 14 (30.4) |

**Supplemental Table 2.** Patient Impressions

| 1 | Completing this study certainly made me more emotional than I thought it would. I think it is great that research is being done on women with CHD and pregnancy. I know I have my own concerns and worries on this topic. I'm sure many other women out there with CHD do as well. I hope this information can provide you all with valuable information to create protocols and guidance for women of childbearing years with CHD going forward.  Having found out that I have a heart condition AFTER having my four children is both fascinating and scary all at the same time. I consider myself fortunate and look forward to sharing information with my adult daughter who also has a congenital heart condition so she can make informed decisions.  I think this is a wonderful study and am willing to help however I can. |
| --- | --- |
| 2 | I have been told to be cautious in terms of pregnancy due to my condition. I have not in the past and am currently not actively trying to become pregnant so I have never been specifically told not to.  I really haven't heard much about it being dangerous for me to get pregnant and I am questioning the seriousness of it and the state of my condition now that it has been brought to my attention.  Thank you for creating this study! It's important to a lot of people with heart conditions to know that they aren't alone and that there are options for them to have children if they want to. |
| 3 | As I get older this question is becoming more important, it's now affecting my daily life and future decisions. The fear in which I've carried with me since childhood about my life being cut short is now officially making me not live my life fully due to more worry than usual-partly surrounding the issue of age my condition and Pregnancy and whether or not I want children / can have them. This wasn't an issue in my 20's but in my 30's I feel the stress now  I feel like women that have congenital heart diseases don't get the same answers from doctors when it comes to pregnancy. We are told from early teen age that getting pregnant can result in higher chances in death than a women with no preexisting conditions. Now as an adult I get told that yes we can get pregnant but we have a 5% of transferring our condition to our baby so there's never just one straight answer when it comes to giving birth. I recently got a new valve and it feels amazing but I worry about the outcome of my unborn child. I worry that he or she will have to go through the same thing I did which has been surgery after surgery all my life. I hope this study can make doctors see that even though we don't ask enough questions about pregnancy because of FEAR we do think about it everyday. Another thing we don't always get a straight answer on is the delivery method (vaginal or c section), I've always been told that if I ever got pregnant I would have it vaginally but my OBGYN's has told me c section so that's another thing that we never get a straight answer. We don't know if to go with our heart doctor or the doctor that is looking after my child and making sure she/ he doesn't have my disease and what's more beneficial for the child outcome.  I would be very curious to know the results of this study and to see how other women with my condition feel about pregnancy and the risks associated. Thank you.  My pregnancy did end up causing complications with my heart. Fortunately he is well and so am I. I got to enjoy seeing him grow up. Now he's married and in the military serving his country. Being a mom was very hard on me. I couldn't of done it, if it wasn't for my support system. Having an infant and not sleeping much was too much for my body. Grandparents stepped up and helped me a lot. Then the stress of teenagers and now the stress of his job. It all takes a toll on my body. I really have to learn how to let stress go and to lean on others to help. There is definitely a long term affect on your heart having kids in a lot to consider. It's not just about what the pregnancy will do to your body |
| 4 | I would love to know what other congenital heart disease females think about this survey. I have always wanted to be a mom and I know a couple of people with congenital heart disease that have given birth so I know it is possible.  REALLY IMPORTANT STUDY, thanks for asking me to participate  Thank you for doing the research study; the question that most threw me was regarding whether doctors had ever recommended that I not get pregnant. I had never been told that directly but every time I asked a medical provider, he/she was visibly uncomfortable and seemingly uncertain as what to say. It always struck me that the more appropriate response would have been to say that he/she didn't know.  This is a great study that I hope provides comfort for women to make a better plan for their future whether they decide to have children or not. I would press that the overall health is taken into account. Some hospitals compartmentalize and waste patient's time and resources, neglecting to provide them with the correct quality of care. Due to this my last child was born in a very traumatic situation that SHOULD and could have been avoided. |
| 5 | … [A] cardiologist told me I can't get pregnant more than 25 years ago. I had c-section because vaginal birth did not work after being in labor for 25 hours  I personally will not be having children. The risks involved sealed the deal when going over the current studies out there and the higher risk factors that would involve me being pregnant. Thank you.  I was referred to a high risk OB/GYN to help determine that I was in fact unable to carry my own children. Because I was unable to be pregnant, I went through a surrogate to have both my children. They are both biologically mine and my husband's children. During IVF and egg retrieval, my heart function was the main concern. I was followed closely by my anticoagulation clinic, my cardiologist, and my high risk fertility doctor. I was admitted to a cardiac floor after my egg retrieval for a week to watch for any abnormal bleeding and or complications. Please feel free to contact me again if you have any more questions or concerns.  Since being told at a young age I can't have my own children, I have grown to be happy with adoption should I want children. Even if I get the "ok" to get pregnant, I'm not sure I actually would want to. I currently do not want children nor see them in my future. |
